# African tick-borne viruses: A systematic review of diversity, epidemiology, clinical impact, and one health strategies

**DOI:** 10.1007/s11259-026-11475-y

**Published:** 2026-08-26

**Authors:** Tsireledzo Goodwill Makwarela, Nimmi Seoraj-Pillai, Tshifhiwa Constance Nangammbi

**Affiliations:** Department of Nature Conservation, Faculty of Science, Tshwane University of Technology, Staatsartillerie Rd, Pretoria West, Pretoria, 0183 South Africa

**Keywords:** Tick-borne viruses, Crimean-Congo haemorrhagic fever virus, African swine fever virus, Jingmen tick virus, One Health, Systematic review, Africa, GRADE, Heterogeneity, CCHFV

## Abstract

**Background:**

Tick-borne viruses (TBVs) are emerging pathogens that affect human and animal health, yet their diversity, epidemiology, and clinical impact in Africa remain poorly synthesised. This systematic review aimed to map TBV diversity, quantify prevalence and seroprevalence, assess clinical outcomes, and evaluate the extent of One Health implementation in surveillance studies across Africa.

**Methods:**

We searched PubMed, Embase, Scopus, Web of Science, CAB Abstracts, African Index Medicus, and grey literature up to 9 April 2026. Independent reviewers screened records, extracted data, and assessed risk of bias using the Joanna Briggs Institute tool for cross-sectional studies. The certainty of evidence was evaluated using GRADE. Heterogeneity was quantified using I², Cochran’s Q, and τ².

**Results:**

Forty-six studies (45 cross-sectional, 1 method development) from 35 African countries were included. Crimean-Congo haemorrhagic fever virus (CCHFV) was the most frequently reported virus (22 studies), followed by African swine fever virus (ASFV, 5 studies) and tick-borne encephalitis virus (TBEV, 3 studies). Novel viruses (Kinna, Bogoria, Perkerra, Ntepes, Shibuyunji, Zaloxo) were also identified. Tick homogenate was the predominant sample type (62.9%). CCHFV minimum infection rates in ticks ranged from 0.13% to 11.3%, while livestock seroprevalence reached up to 98.2% (cattle) and 89.4% (camels). Human CCHFV seroprevalence in at-risk groups ranged from 1.9% to 11.8%, and Kinna virus showed a 38.6% seroprevalence among pastoralists. Only three studies (6.5%) explicitly adopted a One Health framework. Risk of bias was high in 71.7% of studies. Heterogeneity was considerable for CCHFV (I² = 99.9%), ASFV (I² = 99.4%), and JMTV (I² = 97.2%). GRADE certainty was very low for most viruses due to serious bias, inconsistency, indirectness, and imprecision.

**Conclusions:**

CCHFV appears to be widely distributed across Africa based on available evidence, although the certainty of this evidence is very low, particularly given high livestock exposure. The overall evidence is of very low to low certainty. However, 71.7% of studies were at high risk of bias, and GRADE certainty was very low for most viruses, indicating that findings should be interpreted as hypothesis-generating rather than definitive. Novel tick-associated viruses are increasingly detected, yet integrated One Health surveillance remains rare. Future studies should adopt probability-based sampling, confirmatory diagnostics, and multi-sectoral approaches to improve the quality of evidence and inform control strategies.

**Supplementary Information:**

The online version contains supplementary material available at https://doi.org/10.1007/s11259-026-11475-y.

## Introduction

Tick-borne viruses constitute one of the most rapidly expanding segments of arboviral diversity in Africa. They include well-established viruses such as Crimean-Congo haemorrhagic fever virus, African swine fever virus, and tick-borne encephalitis virus, as well as recently detected tick-associated viruses identified through genomic and metagenomic approaches. Recent studies have identified diverse tick-associated viruses, including Alongshan virus, Jingmen tick virus and Beiji nairovirus, while transcriptomic and metagenomic approaches continue to reveal previously undetected viral diversity in field-collected ticks (Brito et al. 2025; Ergünay et al. 2023, 2025; Hoffman et al. 2023; Qin et al. 2022; Quek and Ng 2024; Wikel 2018). This diversity is further shaped by the ecological variety of ixodid and argasid ticks, vertebrate hosts and African biomes (Getange et al. 2025; Portillo et al. 2021).

Several tick-borne viruses cause disease in humans and animals in Africa, with presentations ranging from febrile illness to viral haemorrhagic disease. Crimean Congo haemorrhagic fever virus remains particularly important because exposure has been reported among livestock workers, abattoir personnel and healthcare workers, while surveillance gaps and underdiagnosis continue to obscure the true burden of disease in sub-Saharan Africa (Abdelbaset et al. 2022; Alkhamis et al. 2018; Balinandi et al. 2018; Sazmand et al. 2019; Sui et al. 2025). Geographic expansion of *Hyalomma* ticks, together with climate-driven changes, may further influence exposure risk in affected regions (Chala and Hamde 2021; Ebert and Becker 2025; Perveen and Khan 2022). Animal and wildlife surveillance studies also illustrate the broader importance of integrated monitoring at the human-livestock-wildlife-vector interface (Al-Tayib 2019; Gajda et al. 2022; Newmana and Abrahamsb 2018; Srivastava et al. 2024).

Despite growing evidence, major knowledge gaps remain. First, African data on tick-borne virus diversity, prevalence, and distribution remain uneven across countries and ecological zones (Akinyi et al. 2021; Thoisy et al. 2021). Second, distinguishing true clinical disease burden from incidental exposure or asymptomatic infection remains difficult because of serological cross-reactivity, inconsistent confirmatory testing, and limited linkage between vector surveillance and clinical or seroepidemiological data (Bardhan et al. 2023; Mubemba et al. 2022; Russell et al. 2025). Third, ecological complexity, including migratory birds, livestock movements, climate variability, and habitat change, complicates the prediction of tick-borne virus emergence and spread (Omitola and Taylor-Robinson 2020; Rahman et al. 2020; Recht et al. 2020). These gaps justify a systematic synthesis that separates tick-associated detection from evidence of vertebrate exposure, disease attribution, and One Health implementation.

The One Health paradigm is relevant because tick-borne viruses are maintained within interconnected human, animal, vector, and environmental systems. Multisectoral risk assessment, integrated vector surveillance, abattoir and livestock market surveillance, genomic surveillance and environmental monitoring can improve understanding of spillover risk when applied within coordinated surveillance systems (Borham et al. 2025; Burke et al. 2011; Charles et al. 2021; Moir et al. 2025). Environmental and vector-based surveillance may also contribute to earlier recognition of changing risk patterns by monitoring tick viromes, vertebrate hosts, migratory wildlife, and livestock trade networks (Chauhan et al. 2020; Gondard et al. 2017; Kock and Cáceres-Escobar 2022; Leifels et al. 2022). However, the extent to which studies of African tick-borne viruses have applied integrated One Health approaches remains unclear.

This systematic review synthesises evidence on African tick-borne viruses across four domains: viral diversity and geographic distribution; prevalence and seroprevalence in ticks, animals, and humans; clinical manifestations and disease attribution; and the extent of One Health implementation in surveillance and control studies.

## Methods

### Reporting standards

The search methods and reporting adhere to PRISMA-S. (Li et al. 2021). Two independent reviewers conducted all screening and data extraction steps; disagreements were resolved by consensus or a third adjudicator.

### Eligibility criteria

Studies were included if they addressed tick-associated viruses relevant to Africa, defined as viruses with evidence of tick involvement as a biological vector, reservoir, or part of a maintenance cycle, or viruses detected in ticks within an African context that could be linked to tick ecology rather than to incidental contamination. The geographic scope comprised all African countries and island states; multiregion studies were eligible when African data could be extracted. The prespecified publication window was 1 January 2014 to 9 April 2026. Eligible participants or specimens were humans of any age, vertebrate hosts, and ticks. Primary studies had to report molecular detection, sequencing, virus isolation, serology, or clinically confirmed cases. Purely in vitro studies without field relevance, studies lacking primary viral outcomes, and narrative reviews, editorials, or commentaries were excluded, although their reference lists were screened for eligible primary studies. No language restrictions were imposed; partial extraction from English abstracts was documented when full-text translation was not feasible.

### Information sources

The search was conducted from inception to 9 April 2026 across grouped information sources. Major biomedical databases included PubMed, Embase, Scopus, and Web of Science (Core Collection). Regional databases included African Index Medicus and CAB Abstracts. Grey literature sources included WHO IRIS, Quadripartite One Health guidance documents, Integrated Disease Surveillance and Response technical guidelines for Africa, and WOAH standards on arthropod vector surveillance. Preprint servers included medRxiv, bioRxiv, Research Square and SSRN. Conference proceedings were captured through Embase, Web of Science and Scopus. Reference lists of included studies and relevant systematic reviews were screened, and forward citation searching was performed (Neal R Haddaway et al. 2022a, b). Preprints were clearly labelled as not peer-reviewed.

### Search strategy

Controlled vocabulary (MeSH, Emtree) and free-text keywords were combined for three core concepts: ticks, viruses, and the African geographic context. Truncation, proximity operators, and spelling variants were adapted to each database’s syntax; the full strategies are reported according to PRISMA [3] and were peer-reviewed using the PRESS checklist (McGowan et al. 2016). For robustness, punctuation (e.g., hyphens) was omitted from phrase searches in PubMed, so that “tick-borne” and “Crimean Congo” were searched without dashes.

### Study selection and data management

All retrieved records were imported into RStudio, harmonised and merged into a master dataset. Duplicates were removed using a hierarchical process based on DOI, PMID, the cleaned title, and publication year. Two reviewers independently screened titles, abstracts and full texts using a structured eligibility form. Records were excluded if they were not tick-related, not virus-focused, outside the African context, outside the prespecified publication window, lacked primary data, or lacked a clear tick virus association. Reasons for full-text exclusion were recorded, and final inclusion decisions were retained to support PRISMA reporting and reproducibility.

### Data extraction

During data extraction, tick virus associations were recorded only when a study reported both tick taxonomic identification and virus detection in the same tick species, tick pool or tick-derived sample. We extracted the sample preparation used to establish the association, including whether detection was based on individual ticks, pooled ticks, whole-tick homogenates, blood or serum, tissue samples, or other matrices. We also extracted the diagnostic method used, including RT-PCR, RT-qPCR, nested PCR, antigen detection, ELISA, sequencing, metagenomic NGS, and virus isolation, where reported. Serological findings in vertebrate hosts were not considered direct tick-virus associations unless the same study also tested the identified ticks or tick pools.

### Risk of bias assessment

Design-appropriate tools were applied independently by two reviewers, with consensus and domain-level justifications: the JBI checklist for prevalence studies (Munn et al. 2020); JBI checklists for case series/reports; QUADAS-2 for diagnostic test accuracy studies (Whiting et al. 2011); RoB 2 for randomised trials; ROBINS-I for non-randomised intervention studies (Sterne et al. 2016); SYRCLE for animal experiments; and PROBAST for prediction modelling studies (Wolff et al. 2019). Results informed sensitivity analyses and GRADE certainty ratings.

### Data synthesis

The synthesis comprised four components:

1. **Diversity and ecology mapping**: a catalogue of viruses by taxonomy, tick vectors, vertebrate hosts, and geographic distribution, with evidence maps separating tick-only detections from confirmed vertebrate disease associations.
2. **Epidemiological synthesis**: prevalence and seroprevalence estimates in ticks, humans, and animals. Meta-analysis was considered when ≥ 3 studies were sufficiently homogeneous in terms of design, population, outcome, and diagnostic method. For proportions, variance-stabilising transformations (logit or double arcsine) were used with random-effects models to accommodate expected heterogeneity (Barendregt et al. 2013). Incidence outcomes were synthesised as rates or narratively.
3. **Clinical impact synthesis**: narrative synthesis complemented by structured tables summarising clinical syndromes, severity, hospitalisation, and mortality. Case fatality proportions were pooled under pre-specified comparability criteria, stratified by virus and diagnostic approach.
4. **One Health strategies synthesis**: a narrative, framework-based description of surveillance architectures, laboratory capacity, vector/host control, vaccination, and governance, mapped to the One Health Joint Plan of Action and regional surveillance guidance.

Statistical heterogeneity was assessed using τ² and prediction intervals (Borenstein et al. 2021). Pre-specified subgroups (virus taxon, tick genus/species, host category, African subregion, study period, diagnostic modality, study design) were analysed when subgroups contained sufficient studies for stable estimation (Jpt 2008). Sensitivity analyses excluded studies at high risk of bias, those using low-specificity diagnostic tests likely to inflate prevalence estimates, and outlier or very small studies. When both serological and molecular data were available, analyses were stratified by diagnostic class.

For diagnostic accuracy syntheses, when enough comparable studies existed for a given target condition and index test, bivariate random-effects models were planned to summarise sensitivity and specificity jointly (Reitsma et al. 2005), with hierarchical summary receiver operating characteristic (HSROC) models considered if thresholds varied substantially.

Publication bias and meta-bias were assessed qualitatively (e.g., selective reporting of outbreaks vs. surveillance studies). Funnel-plot-based methods were explored for quantitative syntheses with ≥ 10 studies, acknowledging limitations for analyses of proportional outcomes. For CCHFV, when sufficient extractable estimates were available, exploratory funnel plots were generated from logit-transformed prevalence estimates and their standard errors. The primary funnel plot was restricted to tick molecular detection estimates, while a secondary exploratory plot included all extractable CCHFV estimates. These plots were interpreted with caution because proportional outcomes, heterogeneous sampling designs, different host categories, and variable diagnostic methods may produce asymmetry unrelated to publication bias.

### Certainty of evidence

The GRADE framework was applied by outcome (domains: risk of bias, inconsistency, indirectness, imprecision, publication bias), with explicit justifications (Schünemann et al. 2013). Summary of findings tables were produced for key outcomes with quantitative synthesis; narrative certainty statements were provided for outcomes unsuitable for pooling.

### Reproducibility, ethics, and data management

Screening, extraction, and synthesis were documented using reproducible R-based workflows. Deduplicated records, screening decisions, extraction files, and analytical scripts were retained for verification. Figures were prepared in RStudio and, where stated in the legends, Python 3. Ethics approval was not required because the review used only published and publicly available data.

## Results

### Literature search and screening results

**Fig. 1:**
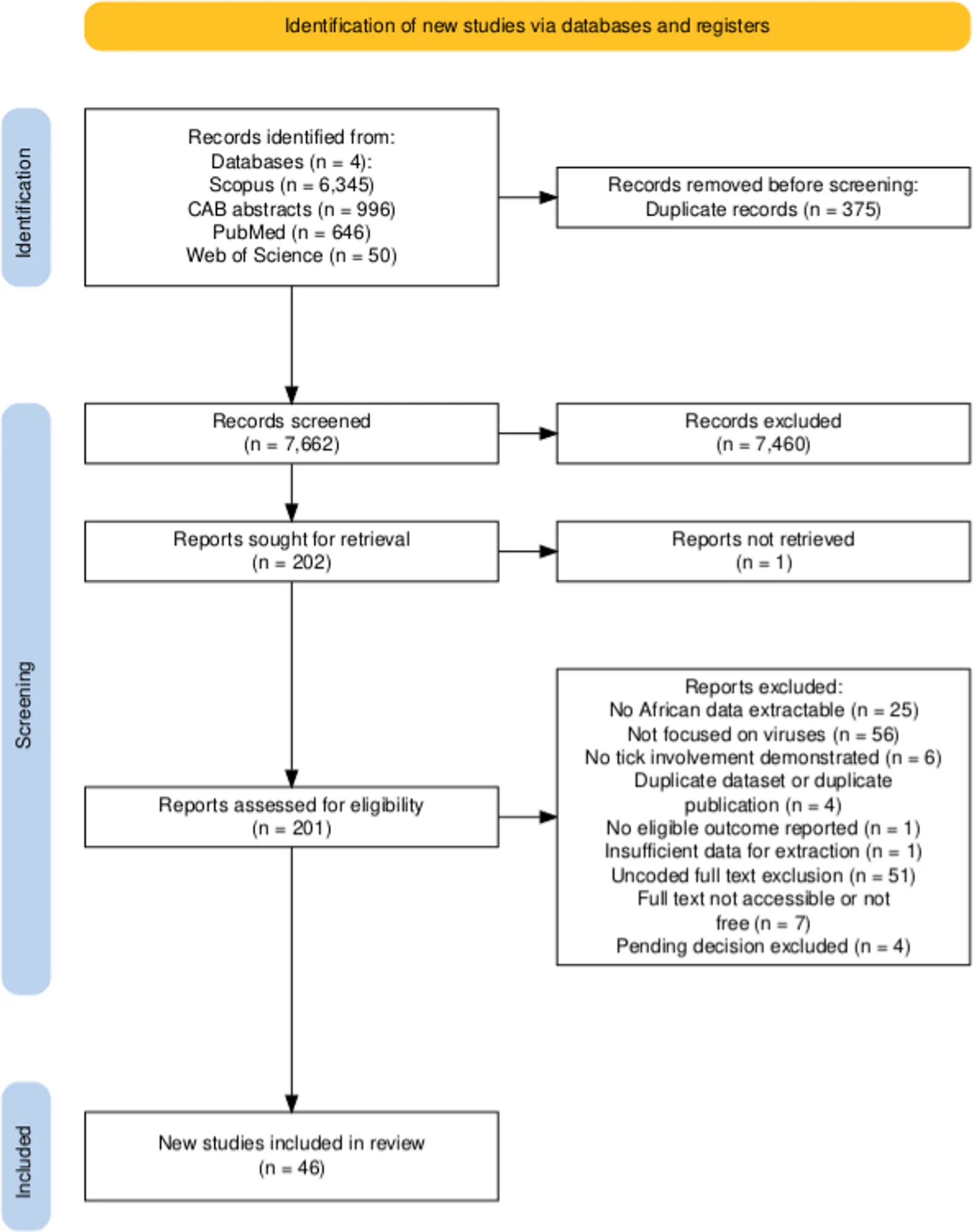
PRISMA 2020 flow diagram. Of 8,037 database records, 7,662 were screened after deduplication, and 46 studies were included in the final review. Reasons for exclusion are shown at each stage. (Neal R. Haddaway et al. 2022a, b)

The systematic search of PubMed, Scopus, Web of Science, and CAB Abstracts yielded 8,037 records. After removing 375 duplicates, 7,662 unique records underwent title and abstract screening, of which 7,460 were excluded (primarily because they were not tick-related, not set in Africa, not virus-focused, or lacking primary data). Full texts of 202 reports were sought, and 78 articles were downloaded for detailed assessment. Fifty-four studies met the initial eligibility criteria; after further review to confirm the presence of viral pathogens, seven studies that focused exclusively on bacteria or parasites were excluded. Consequently, 46 studies were included in the final synthesis (Fig. 1).

### Study characteristics and geographic distribution

**Fig. 2:**
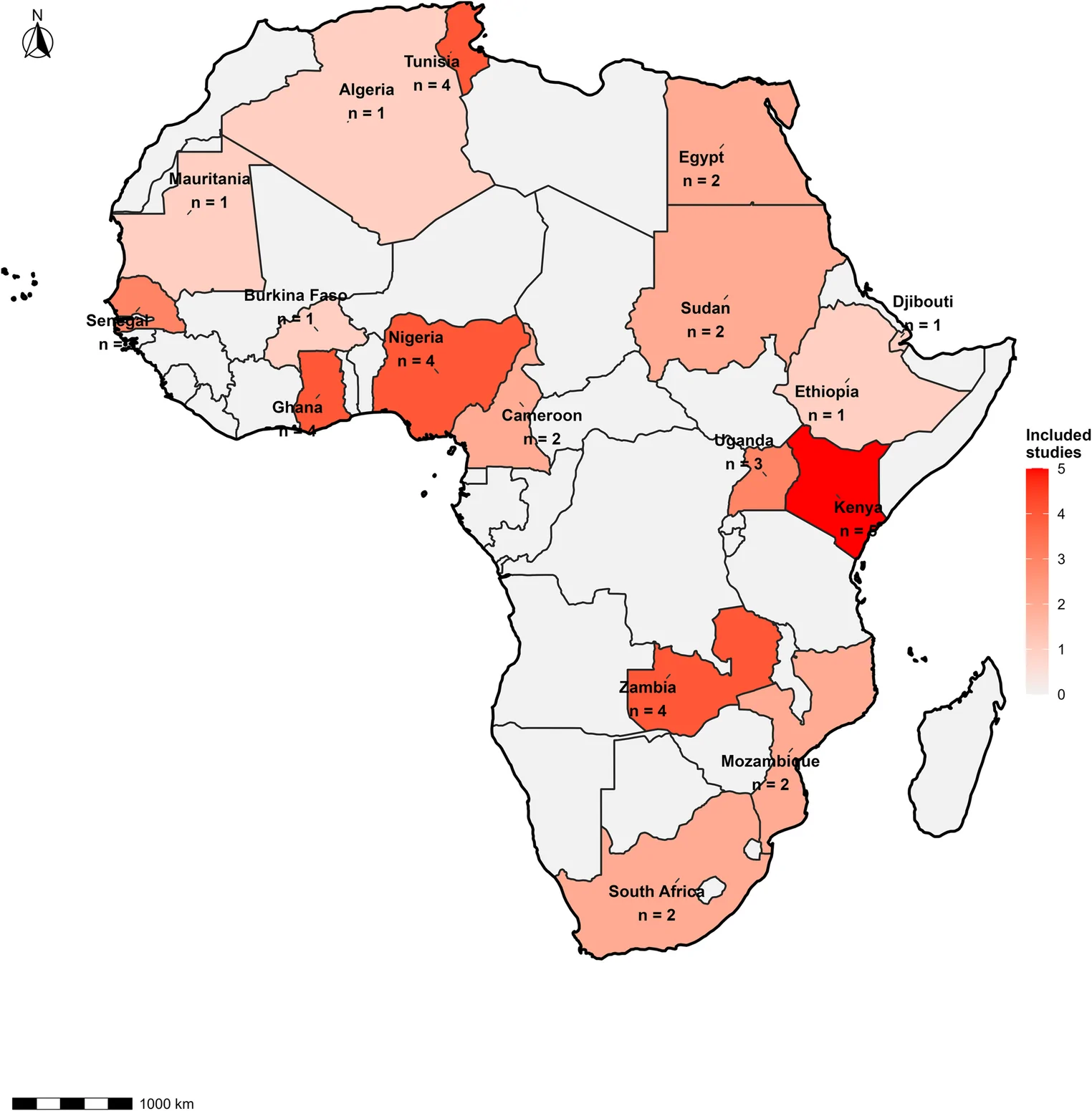
African countries with at least one included study on tick-borne viruses. A total of 35 countries were represented in the systematic review; each shaded country contributed one or more investigations. Map generated in RStudio and refined using Python 3 to enhance country boundaries

**Fig. 3:**
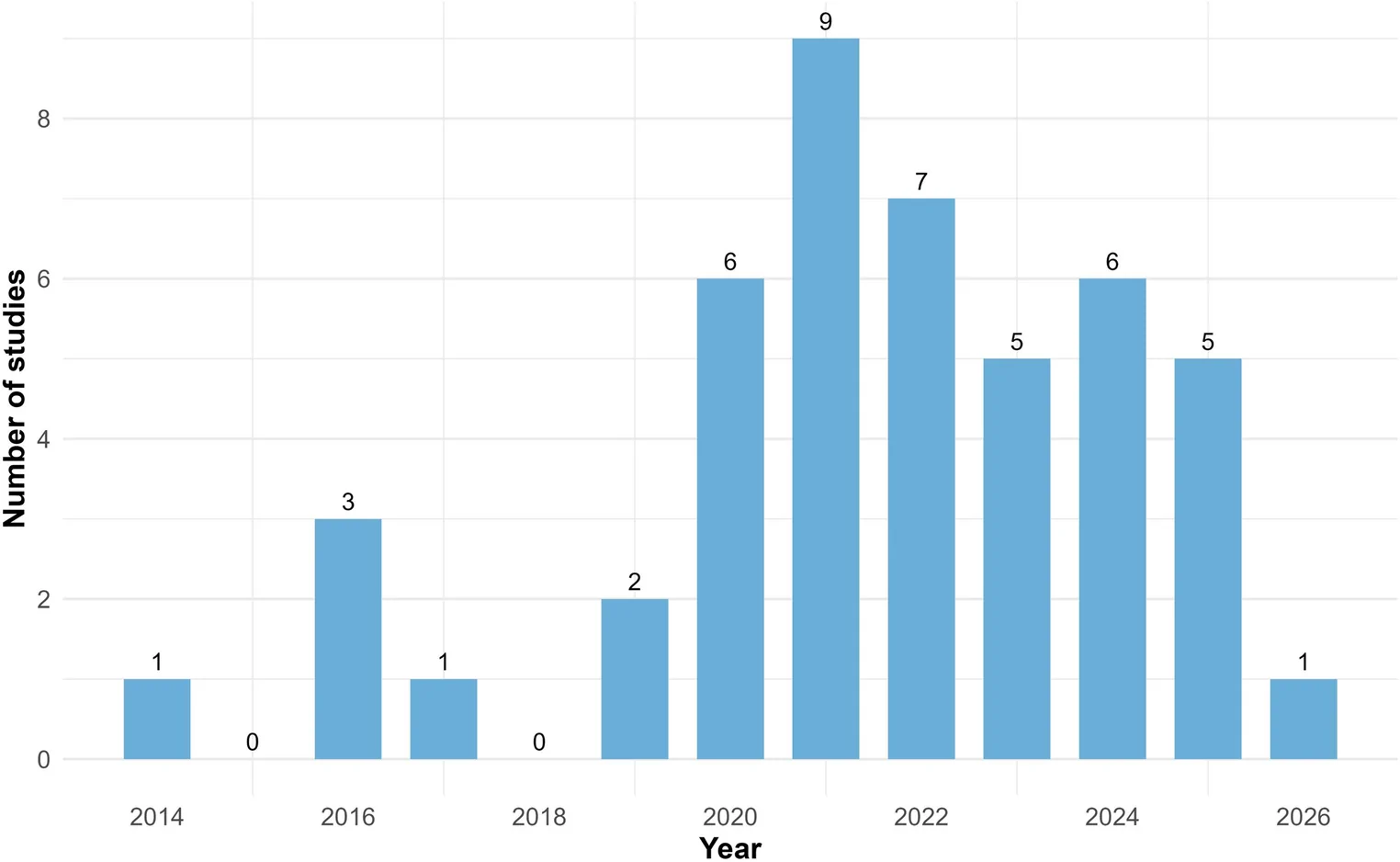
Number of included studies published per year (2014–2026). Research output peaked in 2022 with nine studies, reflecting growing interest in tick-borne viruses—graph generated in RStudio

The 46 included studies, published between 2014 and 2026, covered 35 African countries. Eastern Africa contributed 16 studies, Western Africa 14, Northern Africa 10, Middle Africa 4, and Southern Africa 3; these regional counts were not mutually exclusive because one multicountry study spanned more than one subregion. Kenya contributed five studies, while Tunisia, Ghana, Nigeria, and Zambia contributed four each. Supplementary Table S1 provides author, year, country, virus, design, sample, and virological details. The Democratic Republic of Congo, Chad, and Angola were not represented (Fig. 2). 33 studies (approximately 72%) were published after 2020, with a peak of 9 in 2022 (Fig. 3). Forty-five studies were cross-sectional, and one was a method development study (Schulz et al. 2020).

### Viruses detected

**Fig. 4:**
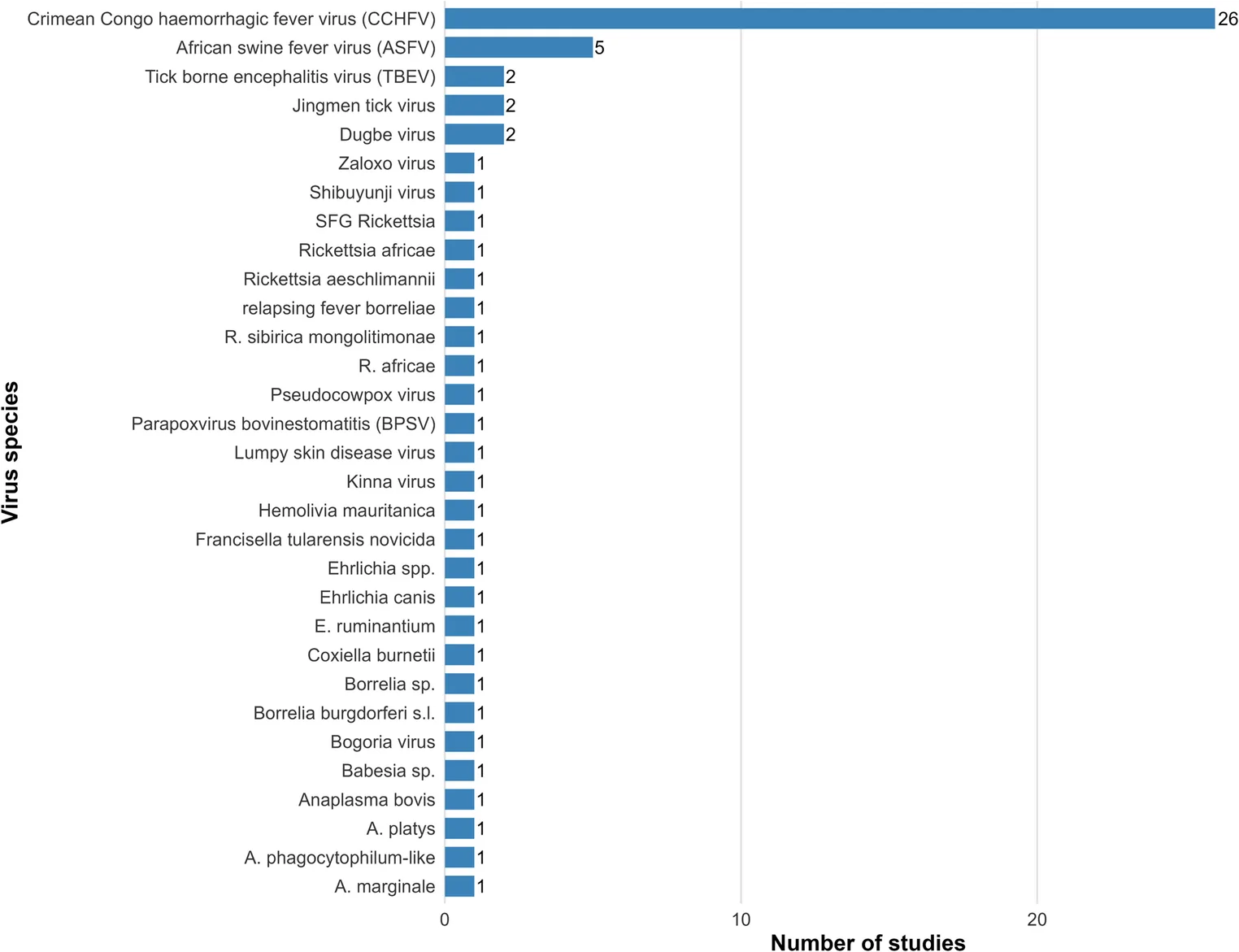
Number of studies investigating each tick-borne virus. Crimean–Congo haemorrhagic fever virus was the most frequently cited in the literature (*n* = 22). Graph prepared using Python 3 and Matplotlib

**Fig. 5:**
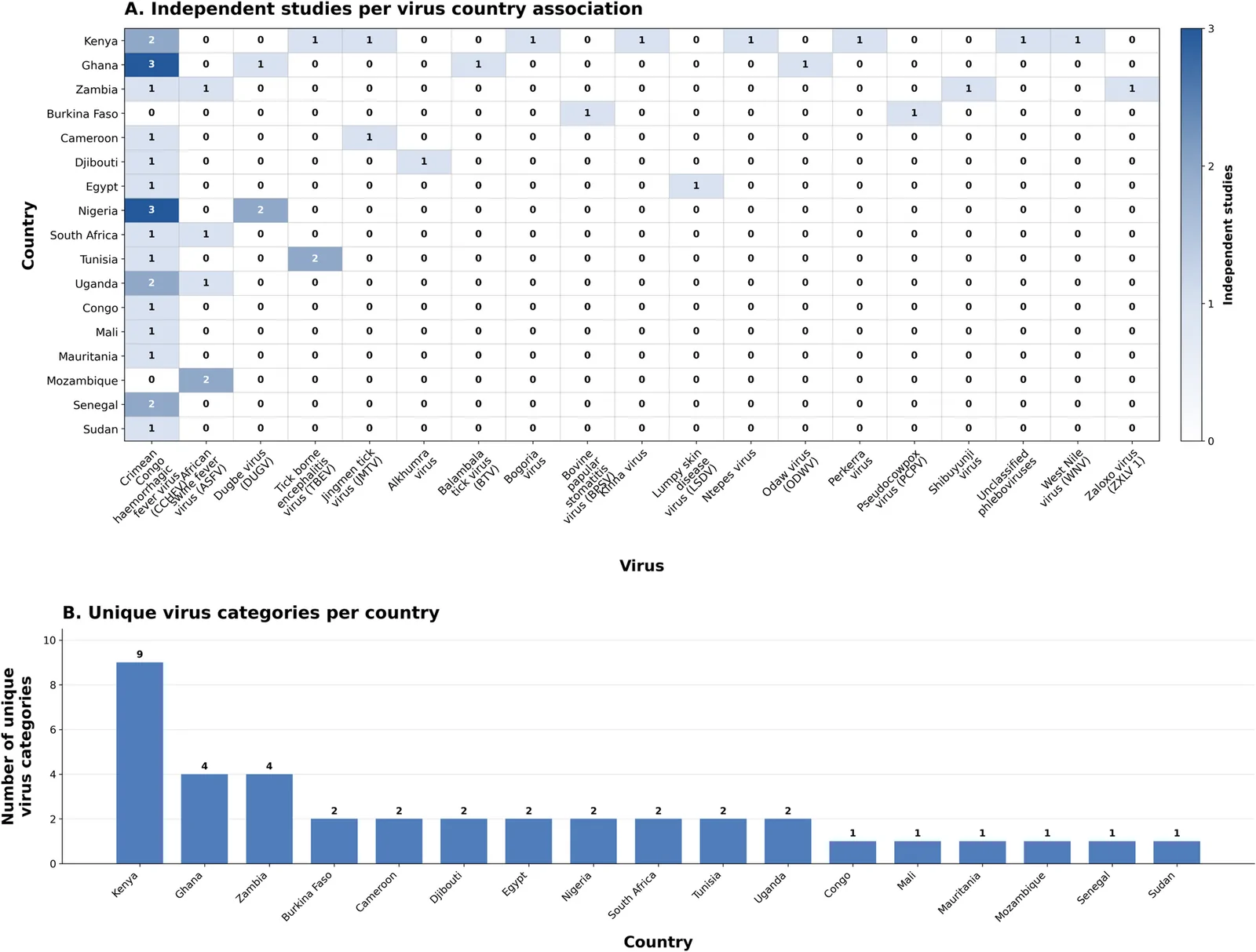
Country-level reporting of tick-borne and tick-associated viruses in Africa. (**A**) Heatmap showing the number of independent studies reporting each virus country association, with darker shading indicating more studies. (**B**) Bar chart showing the number of distinct virus categories reported per country. Blank or white cells in Panel A indicate no positive reports identified in the reviewed literature and should not be interpreted as a confirmed absence of the virus. Panels prepared using Python 3, Matplotlib, and Seaborn

Thirteen viruses met the review’s stricter tick-borne virus classification, while the broader country matrix also retained tick-associated and unclassified viral detections. CCHFV was reported in 22 studies, ASFV in 5, TBEV in 3, and Dugbe orthonairovirus in 2 (Fig. 4). CCHFV was documented in 15 of the 17 countries with extractable virus country data, spanning all five African subregions (Fig. 5). It was detected in *Hyalomma*, *Rhipicephalus*, and *Amblyomma* ticks, and in livestock and human samples; these detections indicate broad reported occurrence but do not, by themselves, establish transmission across all tick genera. ASFV was concentrated in Eastern and Southern Africa and associated mainly with *Ornithodoros* ticks and suid hosts. TBEV was reported from Tunisia and Kenya. Jingmen tick virus pool positivity reached 45.8% in one Kenyan study, and Kinna virus seropositivity was 38.6% in one exposed population. These high study-specific estimates should not be generalised beyond their sampling contexts.

### Tick vectors and sample types

Across the included studies, tick virus associations were most commonly derived from whole tick homogenates or pooled tick homogenates. These preparations were then tested using molecular- or antigen-based methods, most frequently RT-PCR, RT-qPCR, nested PCR, or antigen ELISA, with sequencing or NGS used in some studies to confirm or characterise detections. Fewer studies included virus isolation. Because sample preparation and diagnostic methods varied between studies, the strength of the association between a virus and a specific tick species also varied. Molecular detection in a tick pool indicates the presence of viral nucleic acid in that pool. Still, it does not, by itself, distinguish active tick infection from virus present in a blood meal, environmental contamination, or non-viable viral material.

### Tick vectors and host associations

**Fig. 6:**
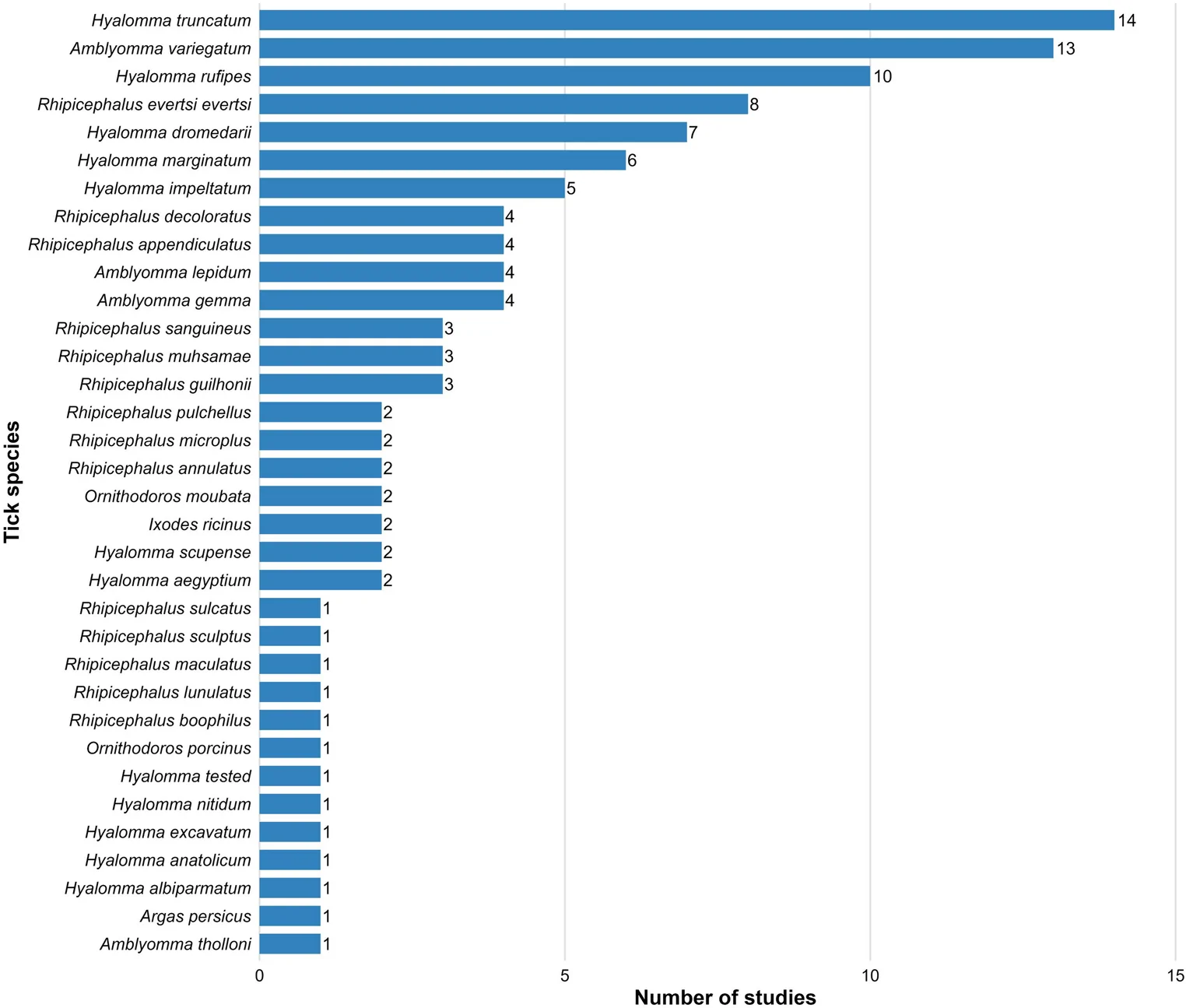
Tick species most frequently reported in the reviewed studies. *Hyalomma rufipes* and *Hyalomma truncatum* were the most common (13 studies each). Graph prepared using RStudio and Python 3

**Fig. 7:**
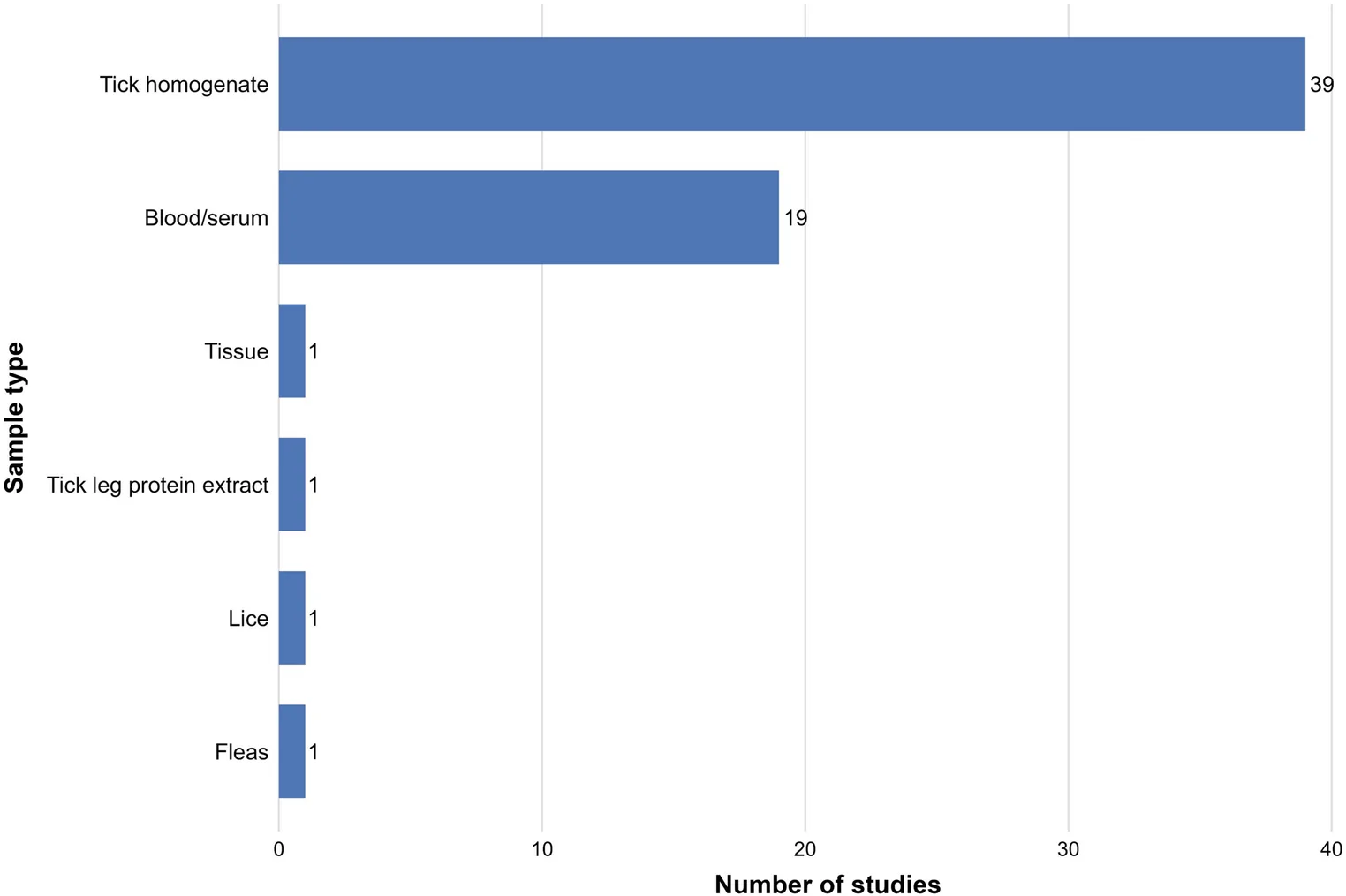
Sample types collected across the included studies. Tick homogenate was by far the most frequently analysed specimen, followed by blood or serum

**Fig. 8:**
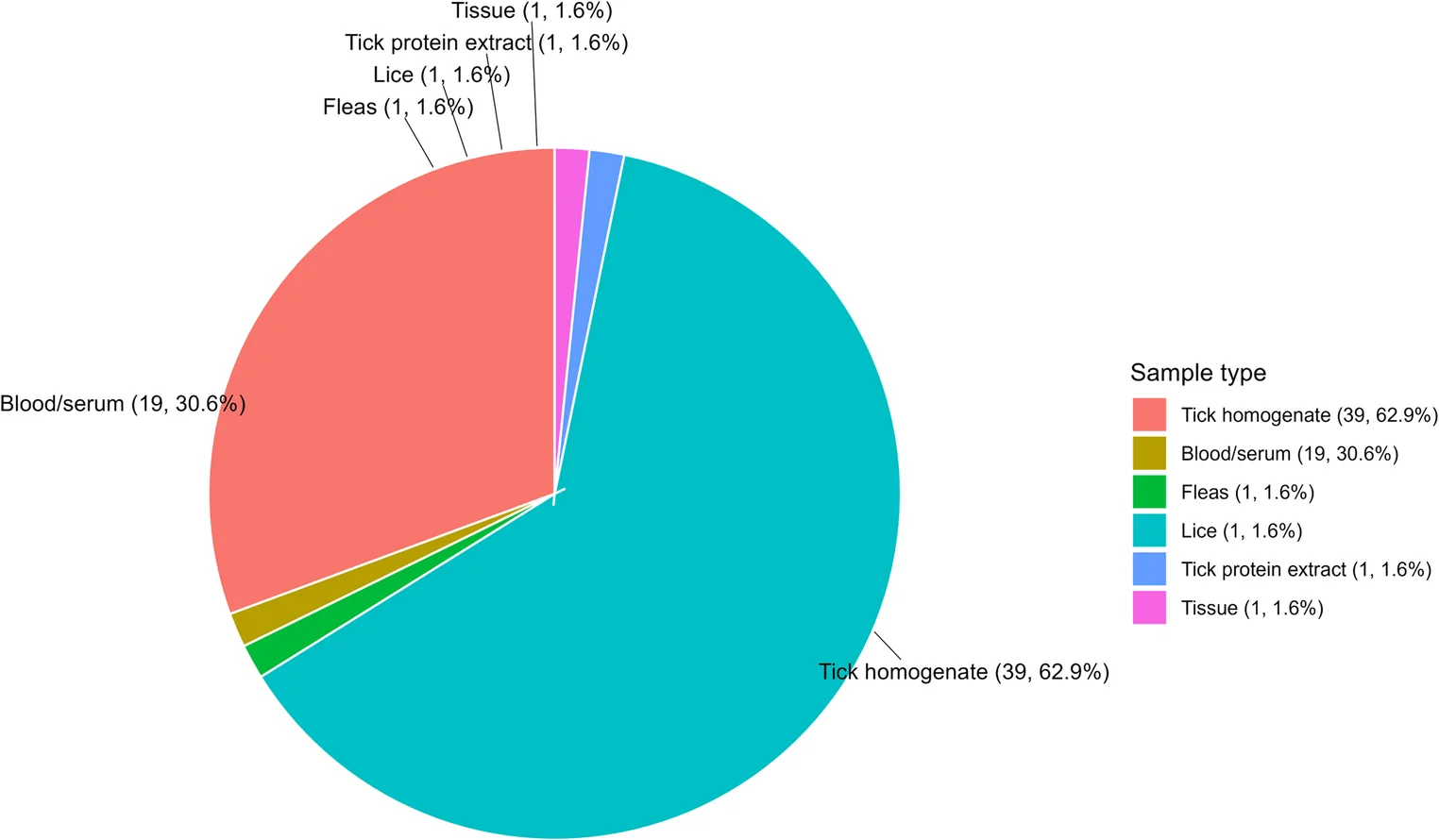
Proportional distribution of sample types based on the total number of mentions, *n* = 62. Tick homogenate was the predominant sample type, accounting for 39 mentions (62.9%), followed by blood/serum with 19 mentions (30.6%). Fleas, lice, tick protein extract, and tissue each accounted for 1 mention (1.6%)

At the species level, *Hyalomma rufipes* and *Hyalomma truncatum* were the most frequently studied vectors (13 studies each), followed by *Amblyomma variegatum* (12 studies), *Hyalomma dromedarii* (7 studies), and *Rhipicephalus evertsi evertsi* (7 studies). Several less common species were also reported, illustrating the broad acarological diversity surveyed (Fig. 6). *Hyalomma* spp. were predominantly linked with CCHFV, *Rhipicephalus* spp. carried both nairoviruses and phleboviruses, and *Amblyomma* spp. were important for the discovery of novel viruses. Regarding sample types, tick homogenate was by far the most common specimen, collected in 39 studies, whereas blood or serum was used in 19 studies. Tissue, tick protein extracts, lice, and fleas were each represented in only one or two studies (Fig. 7). When considering the total number of sample-type mentions (*n* = 62 across all studies), tick homogenate accounted for 62.9% of all collections, and blood/serum for 30.6%, underscoring the heavy reliance on vector-based surveillance (Fig. 8).

### Diagnostic methods

**Fig. 9:**
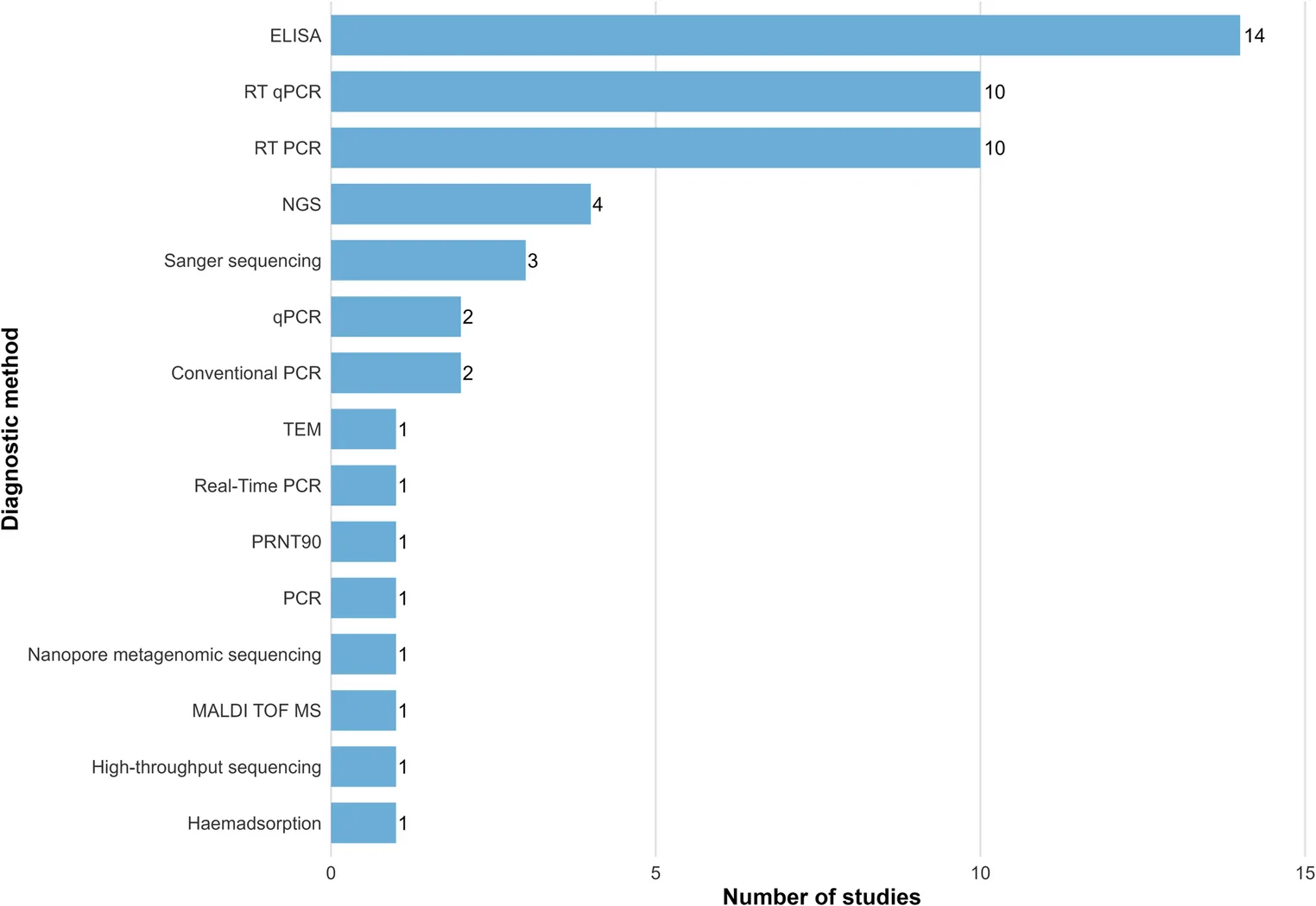
Frequency of diagnostic methods used across the included studies. ELISA, RT-qPCR, and RT-PCR were the most common techniques. Graph prepared using RStudio and Python 3

**Fig. 10:**
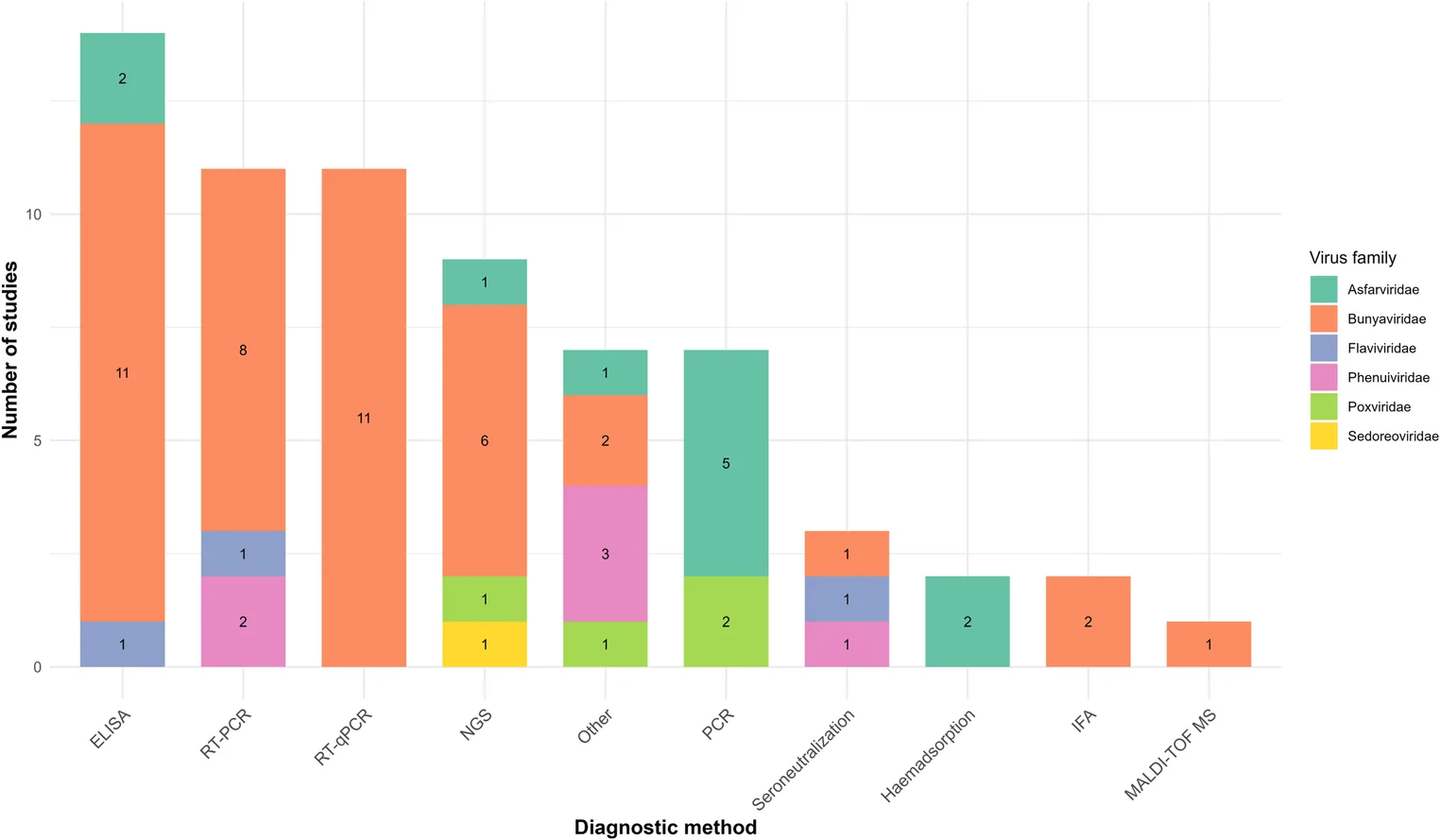
Composition of diagnostic methods by major technique category. Molecular methods dominated; serological approaches were mainly used for seroprevalence surveys. Graph generated in RStudio

Diagnostic approaches varied, with enzyme-linked immunosorbent assay (ELISA) being the most frequently employed method (14 studies), followed by RT-qPCR (11), conventional RT-PCR (11), and next-generation sequencing (NGS; 9). Seroneutralisation, immunofluorescence, haemadsorption, and MALDI-TOF MS were used less frequently (Fig. 9). Grouping the methods into broad categories showed that molecular techniques, PCR, and sequencing were the backbone of virus detection and discovery. At the same time, serological assays were primarily reserved for seroprevalence surveys in humans and livestock (Fig. 10). Sequencing (Sanger or NGS) was performed in 31 studies, reflecting the field’s increasing reliance on genomic data for virus characterisation and phylogenetic inference. Virus isolation was rarely attempted, and proteomic methods remained exceptional.

### Key quantitative findings

Among studies reporting prevalence, tick infection rates were generally low. CCHFV minimum infection rates in tick pools ranged from 0.13% (Senegal) to 11.3% (Mauritania). In contrast, livestock seroprevalence showed wide variation: cattle CCHFV seroprevalence reached 98.2% in Cameroon, 74.2% in South Africa, and 71.9% in Nigeria, while camels in Nigeria and Mauritania displayed rates up to 89.4%. Sheep and goats generally had lower seroprevalences (e.g., 15.6% and 6.6% in Cameroon). Human seroprevalence in at-risk groups ranged from 1.9% to 11.8% for CCHFV, and one study reported 38.6% seropositivity to the novel Kinna virus among pastoralists. No active human outbreaks were recorded; the data represent endemic surveillance rather than outbreak investigations.

### Statistical heterogeneity

Statistical heterogeneity was assessed using Cochran’s Q, I², and the between-study variance τ² for viruses with at least two studies reporting quantitative prevalence data. Q and I² were calculated using inverse variance weights with a 0.5 continuity correction for zero cells. I² values of 25%, 50%, and 75% were interpreted as low, moderate, and considerable heterogeneity, respectively. Four viruses met the criteria for heterogeneity assessment: Crimean Congo haemorrhagic fever virus (CCHFV, 21 studies), African swine fever virus (ASFV, 5 studies), Jingmen tick virus (JMTV, 2 studies), and tick-borne encephalitis virus (TBEV, 2 studies). The corresponding heterogeneity metrics are presented in Table 1. CCHFV showed considerable heterogeneity (I² = 99.9%, Q = 1214.6, df = 20, *p* < 0.001, τ² = 0.00166). Estimates ranged from 0% to 15% in ticks and from 0% to more than 90% in livestock, reflecting substantial differences in geography, hosts, sampling, and diagnostics. ASFV also showed considerable heterogeneity (I² = 99.4%, Q = 650.9, df = 4, *p* < 0.001, τ² = 0.06204), with estimates ranging from 0% in nonoutbreak settings to more than 70% during outbreaks. JMTV showed considerable but unstable heterogeneity (I² = 97.2%, Q = 36.3, df = 1, τ² = 0.00274) because only two studies were available. Reported pool positivity was 45.8% in Kenya and 24.3% in Cameroon. TBEV showed no detectable heterogeneity (I² = 0.0%, Q = 0.0, df = 1, τ² = 0.0), but both estimates were near zero, and statistical power was limited.

**Table 1:** Heterogeneity statistics

Virus species	No. of studies (k)	Cochran’s Q	df	I² (%)	τ²	Interpretation
CCHFV	21	1214.6	20	99.9	0.00166	Considerable heterogeneity
ASFV	5	650.9	4	99.4	0.06204	Considerable heterogeneity
JMTV	2	36.3	1	97.2	0.00274	Considerable (but unstable, k = 2)
TBEV	2	0.0	1	0.0	0.0	No heterogeneity (likely low power)

Abbreviations: ASFV, African swine fever virus; CCHFV, Crimean Congo haemorrhagic fever virus; df, degrees of freedom; I², proportion of total variability attributable to between-study heterogeneity; JMTV, Jingmen tick virus; k, number of studies; Q, Cochran’s Q statistic; TBEV, tick-borne encephalitis virus; τ², estimated between-study variance

#### Narrative synthesis only – heterogeneity precluded meaningful pooling

Extreme heterogeneity for CCHFV, ASFV, and JMTV made a single pooled prevalence inappropriate because it would combine different hosts, sampling frames, diagnostics, and epidemiological settings. Prevalence and seroprevalence were therefore synthesised narratively using study-specific ranges rather than a pooled summary. Subgroup analyses by region, tick genus, host, and diagnostic method were considered but were not reliable. Most subgroups contained too few studies, outcome definitions varied, and 71.7% of studies were at high risk of bias. ASFV studies also mixed outbreak and endemic surveillance, while only two JMTV studies were available. Quantitative estimates are therefore presented in their original study contexts.

### Risk of bias and certainty of evidence

#### Risk of bias within included studies

Two independent reviewers assessed internal validity using the design-appropriate tools specified in Section "Risk of Bias Assessment". Domain-level judgements were resolved by consensus, and each study was classified as having low, moderate, or high risk of bias. Of the 46 studies, 33 (71.7%) were classified as high risk of bias, 11 (23.9%) as low risk, and 2 (4.3%) as moderate risk. The most common sources of bias included convenience sampling (81% of studies), absence of clearly defined inclusion criteria (76%), failure to address confounding (67%), and lack of confirmatory diagnostic methods such as virus isolation or neutralisation assays (44%). In addition, tick-only surveys without concurrent human or livestock sampling were frequent, introducing indirectness, particularly in studies describing novel viruses. Table 2 summarises the risk of bias by virus. CCHFV was reported in 22 studies overall; 21 prevalence studies contributed to the virus-specific risk-of-bias summary and quantitative heterogeneity assessment. Most CCHFV prevalence studies were at high risk because of nonprobability sampling and limited confirmatory testing. ASFV and JMTV had mixed profiles, while a single high-risk study represented several rare viruses.

**Table 2:** Risk of bias summary by virus

Virus species (number of studies)	Low risk	Moderate risk	High risk	Major limitations
CCHFV (21)	4	0	17	Convenience sampling, small pools, no human sera, no confirmatory testing
ASFV (5)	1	1	3	Variable sample sizes; some lacked sequencing
JMTV (2)	1	0	1	Small sample sizes, tick-only surveys
TBEV (2)	1	0	1	Tick-only, low detection
Dugbe orthonairovirus (1)	0	0	1	Single survey, convenience sampling
Shibuyunji virus (1)	0	0	1	Small tick survey, no host sera
LSDV (1)	1	0	0	Large sample, validated methods
Other viruses (each *n* = 1)	0	0	1	Single tick pools, no host data

Abbreviations: ASFV, African swine fever virus; CCHFV, Crimean Congo haemorrhagic fever virus; DUGV, Dugbe orthonairovirus; JMTV, Jingmen tick virus; LSDV, lumpy skin disease virus; TBEV, tick-borne encephalitis virus. CCHFV was reported in 22 studies overall; 21 prevalence studies contributed to this virus-specific summary

#### Assessment of small study effects and publication bias

**Fig. 11:**
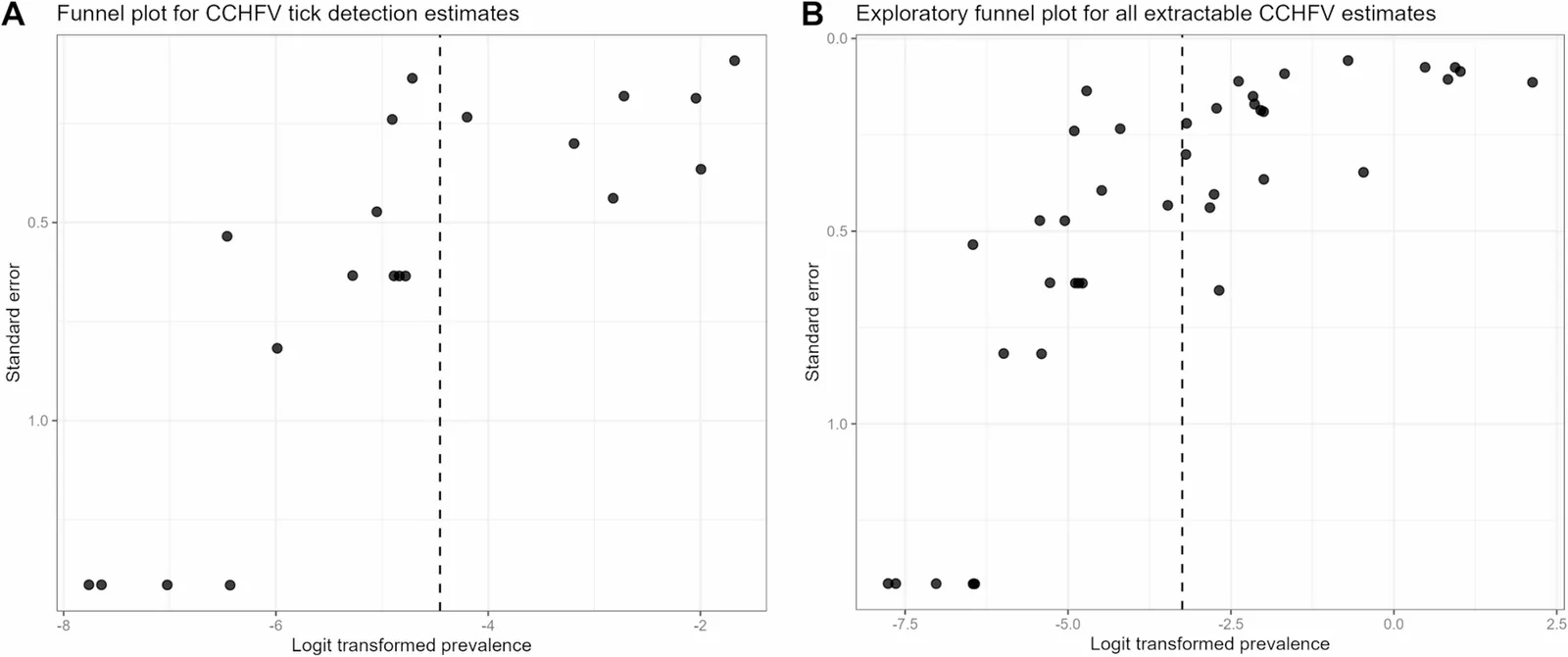
Exploratory funnel plots for CCHFV estimates across included studies. (**A**) Funnel plot for CCHFV tick molecular detection estimates. (**B**) Funnel plot for all extractable CCHFV estimates, including tick, livestock and human observations. Plots generated in RStudio

Exploratory funnel plots were generated for CCHFV because it had the largest number of extractable estimates and was therefore the most suitable virus for visually assessing small-study effects. The primary funnel plot was restricted to tick molecular detection estimates to maintain greater comparability across studies. A secondary exploratory plot included all extractable CCHFV estimates from tick, livestock, and human observations. The tick detection funnel plot showed visible dispersion around the overall effect line, with several less precise studies positioned toward the lower part of the plot (Fig. 11A). The plot, including all extractable CCHFV estimates, showed greater dispersion, reflecting the inclusion of different host groups, sample types, and diagnostic approaches (Fig. 11B). These patterns may indicate small-study effects, selective reporting, or methodological heterogeneity. However, given the high heterogeneity of the evidence base, funnel plot asymmetry should not be interpreted as definitive evidence of publication bias.

#### Certainty of evidence using GRADE

The certainty of the evidence was evaluated using the GRADE framework. Evidence from observational cross-sectional studies was initially considered of low certainty and subsequently downgraded due to risk of bias, inconsistency, indirectness, imprecision, and publication bias. Two reviewers independently assessed each outcome. Table 3 presents GRADE profiles for seven viruses. CCHFV evidence was rated very low due to serious risks of bias, inconsistency, indirectness, and imprecision. ASFV evidence was rated low, while evidence for JMTV, TBEV, Dugbe orthonairovirus, and Shibuyunji virus was rated very low.

**Table 3:** GRADE certainty of evidence

Virus	Studies (*n*)	Risk of bias	Inconsistency	Indirectness	Imprecision	Publication bias	Overall certainty
CCHFV	21	Serious ↓	Serious ↓	Serious ↓	Serious ↓	Possible	Very low
ASFV	5	Serious ↓	Moderate	Serious ↓	Not serious	Possible	Low
JMTV	2	Serious ↓	Serious ↓	Serious ↓	Serious ↓	Unknown	Very low
TBEV	2	Serious ↓	Serious ↓	Serious ↓	Serious ↓	Unknown	Very low
DUGV	1	Serious ↓	NA	Serious ↓	Serious ↓	Unknown	Very low
Shibuyunji	1	Serious ↓	NA	Serious ↓	Serious ↓	Unknown	Very low
LSDV	1	Not serious	NA	Moderate ↓	Not serious	Unknown	Low

Abbreviations: ASFV, African swine fever virus; CCHFV, Crimean Congo haemorrhagic fever virus; DUGV, Dugbe orthonairovirus; GRADE, Grading of Recommendations Assessment, Development and Evaluation; JMTV, Jingmen tick virus; LSDV, lumpy skin disease virus; NA, not applicable; TBEV, tick-borne encephalitis virus; ↓certainty downgraded by one level

LSDV evidence was rated low. Although the single LSDV study used validated diagnostic methods and a large tick sample, it cannot establish biological tick transmission and thus remains indirect for this review question.

### One health and surveillance integration

Only three studies (6.5%) explicitly adopted a One Health framework, although an additional ten studies (21.7%) integrated at least two of human, animal, and vector surveillance components. Vector surveillance was robust across the dataset, whereas human surveillance was limited to 10 studies, and comprehensive environmental surveillance was absent. Tick control strategies were not discussed in any study, and, when present, policy recommendations focused on enhanced cross-border screening, quarantine of imported livestock, and public education. These patterns highlight a significant opportunity to strengthen multi-sectoral collaboration and translate research findings into actionable veterinary and public health interventions.

## Discussion

### Overview of the systematic review

This review synthesised 46 studies from 35 African countries published between 2014 and 2026. CCHFV dominated the evidence base, while ASFV, TBEV, JMTV, phleboviruses such as Odaw and Balambala, Dugbe orthonairovirus, Kinna bandavirus, and several tick-associated poxviruses were also reported. Detection of LSDV, pseudocowpox virus, or bovine papular stomatitis virus in tick-related studies does not establish biological transmission by ticks. These agents were therefore interpreted as tick-associated or incidental detections unless evidence supported vector competence, mechanical transmission, or an epidemiological link.

### Crimean-congo haemorrhagic fever virus: global relevance, principal vectors, and exposure indicators

CCHF is a widely distributed tick-borne viral disease with substantial mortality and no widely available vaccine, making it a priority for public health surveillance worldwide (Ameen et al. 2024; Grech-Angelini et al. 2020; Messina et al. 2015). In our review, CCHFV was the most frequently detected virus across North, West, Central, East, and Southern Africa, reflecting its broad ecological footprint. *Hyalomma* ticks are repeatedly identified as the principal vectors of CCHFV, sustaining enzootic cycles and posing occupational exposure risk to livestock keepers and abattoir workers (Bente et al. 2013; Whitehouse 2004). Our data confirm this pattern: *Hyalomma rufipes* and *Hyalomma truncatum* were the most commonly studied vectors, and CCHFV detections were concentrated in these species, though *Rhipicephalus* and *Amblyomma* ticks were occasionally implicated, consistent with literature noting regional vector variability (Morovvati et al. 2012; Nubgan and Al-Saadi 2023).

Seroprevalence studies in the review consistently showed detectable CCHFV exposure in livestock and humans across Africa, supporting the premise of widespread but heterogeneous circulation (Arteaga et al. 2020; Ebogo-Belobo et al. 2021; Muhammed 2024; Tchetgna et al. 2023). For instance, camel seroprevalence reached 89.4% in Nigeria, and cattle seroprevalence reached 98.2% in Cameroon, yet tick pool PCR positivity rates were considerably lower (e.g., 6% in Uganda). Such discrepancies highlight that past exposure and current infection pressure can diverge, a nuance repeatedly emphasised in reviews (Ameen et al. 2024; Shaker et al. 2024). These findings reinforce the interpretation that CCHF exposure is common in Africa and that integrated surveillance mapping, including both serological and virological endpoints, is necessary to delineate transmission windows and high-risk groups.

### Co-detection, pathogen diversity, and the broader tick virome

Several studies reported co-detection of CCHFV with other viruses or components of the broader tick virome, including Dugbe orthonairovirus, paramyxoviruses, Jingmen tick virus, and Kinna virus. These findings suggest that ticks and their vertebrate hosts may be exposed to multiple viruses within shared ecological settings (Atim et al. 2023; Deézsi-Magyar et al. 2024; Dinçer et al. 2022; Rekik et al. 2024; Zakham et al. 2021). However, co-detection alone does not demonstrate viral reassortment, direct viral interaction, altered transmission risk or vector competence. Evidence for these processes remains limited and would require genomic confirmation, experimental studies and longitudinal surveillance. The detection of Jingmen tick virus and Kinna virus illustrates the expanding diversity of tick-associated viruses in Africa. However, interpretation should remain cautious when the evidence is based mainly on tick pools, metagenomic detection, or limited serological data. Such findings are best viewed as indicators of virome diversity and possible exposure rather than definitive evidence of pathogenicity or transmission competence (Morosan et al. 2023; Yessinou et al. 2025).

### Other tick-borne and animal-associated viruses detected in Africa

Beyond CCHFV, the review captured ASFV, phleboviruses (Odaw, Balambala, Dugbe), and poxviruses (LSDV, pseudocowpox, bovine papular stomatitis virus). ASFV nucleic acid was detected in multiple *Ornithodoros* soft-tick pools in South Africa and in a high proportion of pig sera in Uganda, confirming ongoing sylvatic maintenance and spillover risk. These observations align with the literature, indicating that soft ticks and parapoxviruses detected in cattle represent an extended tick-associated virome, in which mechanical involvement or non-tick transmission pathways may also play roles (Gabriel et al. 2025; Ngnindji-Youdje et al. 2025). The low infection rates observed for phleboviruses and Dugbe virus (~ 0.7% in pools), suggest that these agents circulate at low levels but remain potential causes of febrile illness; their detection in multiple tick genera justifies comprehensive, multi-pathogen surveillance rather than a single-pathogen focus (Amoah et al. 2024; Nepveu-Traversy et al. 2024).

### Ecological and regional drivers of TBV risk

Comparative analyses and reviews emphasise that climate, tick distribution, livestock practices, and cross-border animal movement shape CCHF risk patterns, with climate change potentially altering *Hyalomma* distribution and tick activity windows (Bartolini et al. 2019; Ebert and Becker 2025). Our geographic mapping showed that CCHFV was documented in all five African subregions, and the high seroprevalence in camels and cattle in Sahelian and East African pastoral systems aligns with the recognised role of transhumance and livestock trade in disseminating the virus. The detection of TBEV in Tunisia, albeit at very low prevalence, illustrates how ecological and climatic factors may limit vector distribution while still permitting silent circulation. These observations support prioritising environmental and ecological drivers in risk analyses for CCHFV and other TBVs in Africa (Peintner et al. 2021).

### Surveillance and public health recommendations

The assembled evidence supports strengthening One Health surveillance by linking human febrile illness monitoring, livestock serology, and tick surveillance. Such approaches may improve the interpretation of exposure patterns, particularly in regions affected by livestock movement, transhumance, and cross-border trade (Farès et al. 2016; Whitehouse 2004). Although vector surveillance was relatively common, human surveillance and environmental surveillance were less consistently represented across the included studies. Future surveillance would benefit from coordinated data sharing, standardised diagnostics, and the integration of human, animal, vector, and environmental data (Yessinou et al. 2025). These recommendations should be interpreted in light of the low certainty of the evidence identified in this review. The available data support improved surveillance integration, but they do not yet allow precise estimation of the effectiveness of One Health approaches for tick-borne virus control in Africa.

### Nuances and potential disagreements

Interpretation of the TBV literature requires careful attention to methodological and ecological nuances. *Hyalomma* ticks are consistently highlighted as the principal vectors of CCHFV. Yet, our review and the broader literature document CCHFV RNA in *Rhipicephalus*, *Amblyomma*, and *Dermacentor* ticks, indicating regional variability that necessitates experimental vector competence studies. Moreover, the frequent discrepancy between high seroprevalence in livestock and low tick-pool PCR positivity, as exemplified by our Ugandan data, underscores the importance of multi-endpoint, longitudinal study designs to capture transmission dynamics over time accurately. In one record, an apparent 100% prevalence was due to a proteomic tick identification assay rather than a virological method, highlighting the importance of carefully appraising diagnostic approaches when interpreting prevalence data.

### Comparison with other continents and a global perspective

Global comparisons provide useful context for interpreting African tick-borne virus surveillance, but African findings should be understood within local ecological, veterinary and public health systems. In Europe and Asia, tick-borne encephalitis and severe fever with thrombocytopenia syndrome are more frequently documented, and some regions have established structured surveillance or vaccine-based prevention programmes(Esser et al. 2020; Grech-Angelini et al. 2020; Messina et al. 2015). In contrast, the African evidence base in this review was dominated by CCHFV, ASFV and emerging tick-associated viruses, with limited integration of clinical, veterinary and environmental datasets.

The global relevance of CCHFV is supported by evidence of broad geographic distribution and by the influence of expanding Hyalomma tick distributions, climate variability, and cross-border animal movement on transmission risk (Bartolini et al. 2019; Ebert and Becker 2025; Messina et al. 2015). However, African surveillance priorities should be guided by region-specific factors, including local vector ecology, livestock systems, wildlife interfaces, diagnostic capacity and the level of One Health implementation. Global patterns are therefore useful for comparison, but they should not replace context-specific interpretation of the risk of African tick-borne viruses.

### Limitations and interpretational caution due to high risk of bias

Nonprobability sampling, heterogeneous hosts and outcomes, inconsistent diagnostics, and limited confirmatory testing limited the evidence base. Seropositivity indicates prior exposure, whereas PCR detects viral nucleic acid at the time and place of sampling; these measures are not directly comparable. Detection in whole or pooled tick homogenates confirms the presence of viral material in the tested specimen but does not establish replication, vector competence, transmission, or clinical relevance. The exploratory CCHFV funnel plots were similarly nondiagnostic because host, sample, and method heterogeneity can create asymmetry unrelated to publication bias. RT-PCR and RT-qPCR sensitivity depends on primer design, viral load, preservation, extraction quality, and contamination control. Serology may be affected by cross-reactivity and cannot always separate current from previous infection. Sequencing, metagenomic NGS, neutralisation, and virus isolation provide stronger confirmation but were inconsistently applied. Reported tick virus links should therefore be interpreted as detections or associations unless transmission is demonstrated.

### Certainty of the evidence and implications for future research

Certainty was low to very low for most outcomes because of risk of bias, inconsistency, indirectness, and imprecision. The findings describe reported occurrence and exposure rather than definitive disease burden or transmission intensity. Future studies should use probability-based sampling, confirmatory diagnostics, consistent reporting of hosts, ticks, pools, and targets, and integrated human, animal, vector, and environmental surveillance. Current evidence supports One Health integration as a research and surveillance priority, but does not quantify its effectiveness for virus control.

### Statistical heterogeneity and evidence certainty

Extreme heterogeneity for CCHFV, ASFV, and JMTV, together with sparse data for TBEV, reinforced the low certainty ratings. These statistics reflect differences in hosts, settings, sampling, and diagnostics, and do not support a single pooled prevalence estimate. Standardised protocols and comparable outcome definitions are required before subgroup analysis, or meta-regression can reliably explain regional variation. African tick-borne virus research is expanding, but high risk of bias, limited confirmation, and weak integration across human, animal, vector, and environmental data constrain interpretation. Surveillance should therefore progress from descriptive detection to standardised, confirmatory evidence generation.

## Conclusion

This systematic review indicates that CCHFV is the most frequently reported tick-borne virus in Africa and that diverse additional tick-borne and tick-associated viruses have been detected. Because most evidence was of low or very low certainty, the results should not be interpreted as definitive estimates of burden or transmission. Regional coordination, laboratory capacity, probability-based sampling, confirmatory diagnostics, and integrated One Health surveillance would strengthen future risk assessment and control planning.

## Supplementary Information

Below is the link to the electronic supplementary material.

Supplementary Material 1 (DOCX 32.0 KB)

## Data Availability

No datasets were generated or analysed during the current study.

## Declarations

### Compliance with reporting guidelines

All methods were conducted in accordance with the PRISMA 2020 statement for systematic reviews.
