# Supplementary material for: African tick-borne viruses: A systematic review of diversity, epidemiology, clinical impact, and one health strategies
Source: Vet Res Commun. 2026 Aug 26;50(6):531. doi: 10.1007/s11259-026-11475-y (PMC13518448; doi:10.1007/s11259-026-11475-y)
Supplement: Supplementary file 1 [file 11259_2026_11475_MOESM1_ESM.docx]

**Supplementary Table S1 – Characteristics of included studies (n = 46)**

| **ID** | **Author (Year)** | **Country** | **Subregion** | **Virus(es)** | **Study design** | **Sample type(s)** | **Sample size** | **Key finding (abbreviated)** |
| --- | --- | --- | --- | --- | --- | --- | --- | --- |
| 1 | Laatamna (2022) | Algeria | Northern Africa | CCHFV | Cross-sectional | Tick homogenate | 1043 | All *Rhipicephalus sanguineus* pools negative for CCHFV. |
| 2 | Bendary (2022) | Egypt | Northern Africa | CCHFV | Cross-sectional | Tick homogenate | 25000 | CCHFV Africa I/III lineages detected in *Hyalomma* ticks from imported camels (1.44%). |
| 3 | Schulz (2020) | Cameroon; Mauritania | Middle; Western | CCHFV | Method development | Tick leg protein | 92 | MALDI‑TOF MS reliably distinguishes *Hyalomma* species. |
| 4 | Fares (2021) | Tunisia | Northern Africa | TBEV | Cross-sectional | Tick homogenate | 877 | First TBEV (European lineage) in North Africa (0.11% in *Ixodes ricinus*). |
| 5 | Ogola (2023) | Kenya | Eastern Africa | Bogoria, Perkerra, Ntepes, other phleboviruses | Cross-sectional | Tick homogenate | 4503 | High diversity of novel tick‑associated phleboviruses in Kenyan drylands. |
| 6 | Malonga (2024) | Congo; Mali | Middle; Western | CCHFV | Cross-sectional | Blood/serum | 581 | Low seroprevalence (0.6‑1.75%) in PLWH and blood donors. |
| 7 | Lule (2022) | Uganda | Eastern Africa | CCHFV | Cross-sectional | Blood/serum; tick homogenate | 478 human, 419 cattle, 1065 ticks | Human (10.3%), cattle (69.7%) seroprevalence; CCHFV antigen in *Rhipicephalus*. |
| 8 | Chitimia‑Dobler (2019) | Sudan | Northern Africa | CCHFV | Cross-sectional | Tick homogenate | 598 | First CCHFV Lineage I (Senegal‑like) in *H. impeltatum* in Sudan. |
| 9 | Kajihara (2021) | Zambia | Eastern Africa | CCHFV | Cross-sectional | Tick homogenate; blood/serum | 290 ticks, 1047 cattle | First CCHFV in Zambia; reassortant strain (Africa 3 S, Asia 1/2 M). |
| 10 | Simo Tchetgna (2023) | Cameroon | Middle Africa | CCHFV, Dugbe virus | Cross-sectional | Blood/serum; tick homogenate | 756 serum, 1500 ticks | Very high cattle CCHFV seroprevalence (98.2%). Dugbe in *A. variegatum*. |
| 11 | Kayaga (2024) | Uganda | Eastern Africa | ASFV | Cross-sectional | Blood/serum; tissue | 1328 serum | 37.7% pigs with *O. moubata* tick exposure; linked to ASFV positivity. |
| 12 | Ehizbolo (2024) | Nigeria | Western Africa | CCHFV | Cross-sectional | Blood/serum | 812 | Very high camel seroprevalence (89.4%); risk factors: age, sex, tick burden. |
| 13 | Mhamadi (2022) | Senegal | Western Africa | CCHFV | Cross-sectional | Blood/serum; tick homogenate | 364 human, 34 sentinel sheep, 2238 ticks | CCHFV force of infection in sheep 32%/month; active transmission in Agnam. |
| 14 | Badji (2023) | Senegal | Western Africa | CCHFV | Cross-sectional | Tick homogenate | 6135 | Widespread CCHFV in Senegalese ticks; highest MIR in *B. decoloratus* (6.46/1000). |
| 15 | Msimang (2021) | South Africa | Southern Africa | CCHFV | Cross-sectional | Blood/serum; tick homogenate | 1040 human, 700 cattle, 2376 ticks | Cattle seroprevalence 74.2%; human 3.9% in farm/wildlife workers. |
| 16 | Horton (2016) | Djibouti | Eastern Africa | CCHFV, Alkhumra virus | Cross-sectional | Tick homogenate | 953 | First evidence of both viruses in Djibouti; ALKV in *Amblyomma lepidum*. |
| 17 | Daodu (2021) | Nigeria | Western Africa | Dugbe virus | Cross-sectional | Tick homogenate; blood/serum | 1051 cattle, 11826 ticks | DUGV detected in 0.7% ticks (mostly *A. variegatum*); cattle sera negative. |
| 18 | Daodu (2024) | Nigeria | Western Africa | CCHFV, Dugbe virus | Cross-sectional | Blood/serum | 877 (CCHFV), 854 (DUGV) | High seroprevalence: CCHFV 71.9%, DUGV 52.8%; 37.9% co‑exposed. |
| 19 | Koka (2024) | Kenya | Eastern Africa | Kinna virus (novel) | Cross-sectional | Tick homogenate; blood/serum | 2342 ticks, 324 humans | Novel bandavirus; high human seroprevalence (38.6%); lethal to mice. |
| 20 | Daodu (2025) | Nigeria | Western Africa | CCHFV | Cross-sectional | Tick homogenate; blood/serum | 318 cattle sera, 2855 ticks | CCHFV in *H. truncatum* pools; sequences similar to IbAr10200. |
| 21 | Mwasi (2025) | Kenya | Eastern Africa | JMTV | Cross-sectional | Tick homogenate | 1629 (72 pools) | JMTV detected in 45.8% tick pools; evidence of recombination and selection. |
| 22 | Ouedraogo (2020) | Burkina Faso | Western Africa | PCPV, BPSV | Cross-sectional | Tick homogenate | 663 (171 pools) | First detection of parapoxviruses in West African ticks. |
| 23 | Wampande (2021) | Uganda | Eastern Africa | CCHFV | Cross-sectional | Tick homogenate | 70 | CCHFV genotype II in *R. decoloratus*; novel M segment diversity. |
| 24 | Mhamadi (2022) | Senegal | Western Africa | CCHFV | Cross-sectional | Tick homogenate; blood/serum | 2238 ticks, 1317 sheep, 335 human | Multiple CCHFV genotypes co‑circulating in ticks and sheep. |
| 25 | Ndlovu (2020) | South Africa; Zambia | Southern Africa | ASFV | Cross-sectional | Tick homogenate | 3 isolates | Draft genomes of three ASFV isolates from *Ornithodoros* ticks. |
| 26 | Amoa‑Bosompem (2021) | Ghana | Western Africa | Odaw, Balambala, Dugbe | Cross-sectional | Tick homogenate | 354 (93 pools) | Odaw (dogs), Balambala and Dugbe (cattle) detected. |
| 27 | Nyataya (2020) | Kenya | Eastern Africa | WNV, TBEV, CCHFV | Cross-sectional | Blood/serum | 1033 | Serological evidence of exposure; highest CCHFV (5.2%) in Lodwar. |
| 28 | Craig (2021) | South Africa | Southern Africa | ASFV | Cross-sectional | Tick homogenate | not specified | First report of *O. zumpiti* in warthog burrows; no ASFV detected. |
| 29 | Wasfi (2016) | Tunisia | Northern Africa | CCHFV | Cross-sectional | Blood/serum; tick homogenate | 181 humans, 38 slaughter workers, 310 ticks | First serological evidence of CCHFV in Tunisia; no virus in ticks. |
| 30 | Fares (2019) | Tunisia | Northern Africa | CCHFV | Cross-sectional | Tick homogenate | 1174 | No CCHFV in *H. aegyptium* from tortoises; high tick infestation (91%). |
| 31 | Shuaib (2020) | Sudan | Northern Africa | CCHFV, *Rickettsia* | Cross-sectional | Tick homogenate | 1593 | *Rickettsia aeschlimannii* and *R. africae* detected; no CCHFV RNA. |
| 32 | Simulundu (2021) | Zambia | Eastern Africa | Shibuyunji virus | Cross-sectional | Tick homogenate | 363 | Novel phlebovirus in 12% of *Rhipicephalus* ticks from Namwala. |
| 33 | Chadd (2025) | Ethiopia | Eastern Africa | Multiple bacteria & BPSV | Cross-sectional | Tick homogenate | 26218 (154 pools) | First metagenomic survey; detected *Coxiella*, *Rickettsia*, *Francisella*, BPSV. |
| 34 | Kiwan (2025) | Cameroon | Middle Africa | JMTV | Cross-sectional | Tick homogenate | 622 (251 pools) | First JMTV in Central Africa; high prevalence in *R. microplus*. |
| 35 | Chusyd (2025) | Zambia | Eastern Africa | Zaloxo virus (novel) | Cross-sectional | Tick homogenate | 6 | Novel orbivirus from elephant tick *A. tholloni*; pathogenicity unknown. |
| 36 | Akuffo (2016) | Ghana | Western Africa | CCHFV | Cross-sectional | Tick homogenate; blood/serum | 144 ticks (97 pools), 108 humans | CCHFV in ticks and abattoir workers (human seroprevalence 5.7%). |
| 37 | El Ghassem (2023) | Mauritania | Western Africa | CCHFV | Cross-sectional | Blood/serum; tick homogenate | 263 humans, 1380 animals, 282 ticks | Widespread CCHFV; camel (89.4%) and cattle (74.6%) seroprevalence. |
| 38 | Khamassi Khbou (2020) | Tunisia | Northern Africa | TBEV | Cross-sectional | Blood/serum | 263 sheep | First TBEV serological evidence in North Africa (sheep, SNT‑confirmed). |
| 39 | El Ansary (2022) | Egypt | Northern Africa | LSDV | Cross-sectional | Tick homogenate | 4000 (20 pools) | First molecular evidence of LSDV in *R. annulatus* ticks; virus isolated. |
| 40 | Makayi (2026) | Zambia | Eastern Africa | *Anaplasma* spp. | Cross-sectional | Tick homogenate | 588 | Detected *Anaplasma* in ticks from cattle, buffalo, tortoise. |
| 41 | Seth‑Addo (2024) | Ghana | Western Africa | CCHFV | Cross-sectional | Tick homogenate | 1550 (491 pools) | First *Hyalomma marginatum* in Ghana; CCHFV reassortant. |
| 42 | Bentil (2023) | Ghana | Western Africa | CCHFV | Cross-sectional | Tick homogenate | 705 | First whole‑genome sequencing of CCHFV in Ghana (genotype III). |
| 43 | Quembo (2014) | Mozambique | Eastern Africa | ASFV | Cross-sectional | Blood/serum; tick homogenate | 12 warthogs, 629 pigs, 20 pens | First evidence of sylvatic ASFV cycle in Mozambique. |
| 44 | Norte (2022) | Morocco, Algeria, Tunisia | Northern Africa | *Rickettsia*, *Borrelia*, *Hemolivia* | Cross-sectional | Tick homogenate | 131 | High pathogen diversity in *H. aegyptium* from tortoises. |
| 45 | Quembo (2017) | Mozambique | Eastern Africa | ASFV (genotypes II, V, XXIV) | Cross-sectional | Tick homogenate | 1662 | Confirmed sylvatic cycle; 19 isolates including new genotype XXIV. |
| 46 | Chiuya (2021) | Kenya | Eastern Africa | CCHFV, *Rickettsia*, *Anaplasma*, *Theileria*, *Babesia* | Cross-sectional | Tick homogenate; lice; fleas | 486 arthropods (333 pools) | CCHFV in *Rhipicephalus decoloratus*; high *R. africae* prevalence. |
